# Motor speed does not impact the drift rate: a computational HDDM approach to differentiate cognitive and motor speed

**DOI:** 10.1186/s41235-022-00412-7

**Published:** 2022-07-22

**Authors:** Joshua Sandry, Timothy J. Ricker

**Affiliations:** 1 Psychology Department, Montclair State University, 1 Normal Ave, Montclair, NJ 07043 USA; 2 Department of Psychology, University of South Dakota, 414 E. Clark Street, Vermillion, SD 57069 USA

**Keywords:** Reaction time, Sequential sampling model, Model validity, Motor speed, Information processing speed

## Abstract

The drift diffusion model (DDM) is a widely applied computational model of decision making that allows differentiation between latent cognitive and residual processes. One main assumption of the DDM that has undergone little empirical testing is the level of independence between cognitive and motor responses. If true, widespread incorporation of DDM estimation into applied and clinical settings could ease assessment of whether response disruption occurs due to cognitive or motor slowing. Across two experiments, we manipulated response force (motor speed) and set size to evaluate whether drift rates are independent of motor slowing or if motor slowing impacts the drift rate parameter. The hierarchical Bayesian drift diffusion model was used to quantify parameter estimates of drift rate, boundary separation, and non-decision time. Model comparison revealed changes in set size impacted the drift rate while changes in response force did not impact the drift rate, validating independence between drift rates and motor speed. Convergent validity between parameter estimates and traditional assessments of processing speed and motor function were weak or absent. Widespread application, including neurocognitive assessment where confounded changes in cognitive and motor slowing are pervasive, may provide a more process-pure measurement of information processing speed, leading to advanced disease-symptom management.

## Significance statement

Neurological disorders may lead to both cognitive and physical disability, for example, slowing of information processing speed and/or slowing of motor function, respectively. Unfortunately, many commonly used neurological measures that are designed to evaluate cognitive changes do not account for related changes in physical functioning. That is, patients thinking may be evaluated with a timed assessment that requires them to press buttons on a computer keyboard or write down responses on a sheet of paper. In this context, a low score would be interpreted as a slowing of information processing speed. While these assessments may capture changes in information processing speed, they would also reflect any comorbid changes in motor slowing. This mismeasurement is problematic because it provides an unclear picture of cognitive and physical disability and presents a serious challenge in understanding *and* eventually treating cognitive disability due to neurological disorders. This study suggests that computational modelling approaches can be used to differentiate slow information processing speed (cognitive) from slow motor speed (physical). This will help improve measurement precision of disease-related changes, especially when applied to neurological conditions where both cognitive and motor functioning are negatively impacted.

## Introduction

People make many decisions that vary in complexity on a daily basis and often these decisions occur under time pressure. In order to understand the cognitive processes that underlie decision making, researchers have increasing applied the drift diffusion model (DDM), a sequential sampling model, in both basic and clinical research (Evans & Wagenmakers, 2019; Ratcliff et al., 2016; White et al., 2010). The DDM is a binary evidence accumulation model that incorporates response time and accuracy to decompose behavioral data into parameter estimates that represent latent cognitive processes. The latent cognitive processes reflect the decision and non-decision aspects that underlie decision making.

The primary components of the DDM include drift rate (*v*), boundary separation (*a*), and non-decision time (*Ter*). The drift rate parameter reflects the rate of information processing where evidence is accumulated stochastically until some threshold or boundary is reached. Acquiring more evidence over time pushes the drift rate toward a decision boundary. The boundary separation parameter is an estimate of response caution or the degree of conservative responding and reflects the tradeoff between speed and accuracy. The non-decision time parameter reflects the residual aspects of the decision process including pre-stimulus encoding (Te) and motor response execution (Tr). Together, the residual encoding and motor response are jointly modelled by the *Ter* parameter. In more complex instantiations of the full DDM, additional parameters include estimates of bias along with across-trial variability associated with the primary DDM parameter estimates (Ratcliff & Tuerlinckx, 2002). While existing research supports this interpretation of the drift rate and boundary separation parameters (Forstmann et al., 2016; Ratcliff & McKoon, 2008; Ratcliff et al., 2016), little research has directly investigated the degree that changes in motor speed may influence the drift rate (*v*). It is typically assumed that motor changes should only influence the motor execution parameter (*Ter*). We discuss these papers subsequently but first discuss the need for and limitations of applying the DDM clinically.

## Clinical potential and limitations of the DDM

There is a strong need for more precise measurement in clinical research and this is one area where application of the DDM has strong potential (Evans & Wagenmakers, 2019). In some neurological and psychiatric diseases, slowing of information processing speed is a common finding. However, the psychometric validity of this conclusion is somewhat tenuous. Often, processing speed inferences are based on murky cognitive assessments that tap into much more than “speed” alone (Sandry et al., 2021). For example, commonly used digit-symbol coding tests require participants to match symbols in a grid with corresponding digit-symbol pairings in a key in a 90 s time period. The number of correct responses is inferred as an estimate of information processing speed. The problem is that, along with a speeded component, these assessments also tap into a wide range of other cognitive processes including learning and memory, language, visual search/attention, etc. (Jaeger, 2018; Joy et al., 2003; Sandry et al., 2021; Treviño et al., 2021). In this context, low test scores could reflect changes in cognitive speed, or they may reflect changes in some other cognitive, perceptual or sensorial process. As a result, the clinical description may be mischaracterized, obfuscating our understanding of cognitive change and leading to challenges for treatment.

The drift rate parameter may be a more accurate reflection of disease-related changes in information processing speed than traditional amorphous neuropsychological assessments. Importantly, motor slowing is comorbid with cognitive change in many neurological conditions, introducing additional psychometric challenge. For example, Parkinson’s disease, brain injury, stroke, cancer, and multiple sclerosis all may experience varying degrees of physical and cognitive disability. Therefore, it is critical to directly test whether any effect of motor slowing is captured by the drift rate. If drift rate is uncontaminated by motor processes, this would provide some confidence in using the DDM to guide clinical decision making for patient populations with confounded motor and cognitive slowing.

### DDM and motoric processes

Despite the importance of the assumption that drift rate is free from motoric influences for interpretation of empirical and clinical data, there are only a small number of studies that have investigated motor effects on the DDM in general. We are aware of only a few investigations that have included experimental manipulations of motor speed slowing and the impact on DDM parameters. The outcomes and conclusions of these investigations are somewhat mixed. This is partially a result of design limitations, as the primary research questions are not always aimed at understanding motor slowing effects on the drift rate parameter. This is the primary aim of the current investigation.

In one of the first investigations of how the theoretical DDM parameters map onto empirical data, Voss et al. (2004) included a direct manipulation of motor response with a “response handicap condition”. In this condition, participants used the same finger to respond in a color discrimination task. Their response finger was positioned centrally on the B key of the keyboard, between the C and M response keys.^1^ This manipulation added additional motor travel time to the participants response. Importantly, this travel time manipulation may differ from a motor slowing manipulation. In the critical comparison, the response handicap condition led to larger values of non-decision time, suggesting some support for this parameter reflecting motor processes. The authors also reported a decrease in drift rate (*v*) for the response handicap (motor travel) condition in comparison with the non-handicap condition, which may imply drift rate is not immune to the effect of motor slowing.

Similar findings of motor speed impacting drift rate were subsequently reported by the same group. Specifically, participants were asked to make a stimulus keypress response three times in a row for each trial. Response accuracies and latencies were collected for each keypress to estimate three DDM’s and evaluate changes in non-decision time along with other DDM parameters, across each of the three sequential responses. The findings showed the expected effect of longer non-decision times for the second in comparison with the first key press. The keypress manipulation also resulted in unexpectedly smaller drift rates and lower boundaries. In fact, the effect on drift rates was larger than the effect on non-decision time. This may imply the DDM parameters are either indiscriminate or that the experimental manipulation of motor speed was invalid (Lerche & Voss, 2019). In contrast, Gomez et al. (2015) compared drift rates as a function of three response modalities: eye-movements, key-presses and a touchscreen display and reported no differences in drift rate as a function of response modality.

Other investigations have applied alternative approaches to evaluate the relationship between motor speed and DDM parameter estimates. For example, Weindel et al. (2021a) used electromyograph (EMG) to record neurophysiological activation of the effector responding muscle and mapped this measurement onto the latent process of non-decision motor response time. This design allowed encoding time (*Te*) to be partitioned from response execution time (*Tr*). Across two experiments, the authors reported that within participant manipulations of perceptual difficulty along with manipulations of speed and accuracy both impacted the response execution/motor time component of *Ter*. Additionally, the authors included a between-experiment manipulation of response force. The force threshold required to depress the key and record the response required less force in experiment 2 in comparison with experiment 1. Somewhat unexpectedly, the analysis of non-decision time across the two experiments revealed no difference as a function of the response force manipulation with nearly identical parameter estimates. An additional finding beyond evaluating non-decision time that was not discussed, and particularly relevant to the present research, was that the between-experiment manipulation of response force may have also led to changes in drift rate. The interpretation of the motor speed manipulation on non-decision time is limited because the between experiment manipulation was not optimized to evaluate the impact of response force on drift rate. There were other subtle design changes across experiments 1 and 2 that introduce additional challenges for a meaningful and unbiased comparison, for example, a change in the difficulty level across experiments. These differences render it challenging for direct comparison across experiments and whether or not we would expect to observe any differences in drift rate as a function of the motor speed manipulation. It is reasonable to suppose that a within participant experimental manipulation of response force would cause expected changes in non-decision time and permit a reliable evaluation of motor speed effects on drift rates.

## Present experiments

In the present research, we contrast an *Independence Hypothesis*, whereby changes in motor speed will impact non-decision times but they will not impact drift rates against a *Non-Independence Hypothesis*, whereby changes in motor speed will impact both non-decision times and drift rates. Specifically, under optimized design conditions, absence of an effect of motor response force on drift rate with presence of an effect of motor response force on non-decision time would provide strong evidence in favor of an *Independence Hypothesis*. Alternatively, under the same conditions, presence of an effect of motor response force on drift rate with presence of an effect of motor response force on non-decision time would provide strong evidence in favor of a *Non-Independence Hypothesis*. If verified, data in favor of the *Independence Hypothesis* would provide compelling evidence to continue to integrate the DDM analytic approach into clinical research and practice. We aim to fill this gap in the present investigation by directly manipulating (1) motor response force by changing the spring pressure in customized response button boxes and (2) set size by varying the number of stimuli presented to participants. These manipulations allow us to observe how DDM parameter estimates may change across the full factorial of experimental conditions. We note that independence in the context of the present investigation is operationalized as measurement independence between evidence accumulation (central nervous system) and *physical* motor execution *measured at the hand* (peripheral nervous system). This differs from other theoretical conceptualizations of independence that differentiate between more than one motor component. For example, a *cognitive* premotor planning/initiation component along with *physical* motor speed. That is, fractionating non-decision time, and specifically *Tr*, into premotor planning + motor time (Servant et al., 2021). We focus our manipulations and questions specifically on whether *physical* motor slowing is or is not captured by the drift rate (*v*) parameter.

### Modelling hypothesis

On the basis of diffusion model assumptions, available literature, and manipulations in the present study, we made the following primary modelling predictions. Specifically, given larger set sizes should require more cognitive resources, we expected an effect of Set Size to change the drift rate. Additionally, given the larger set size includes more letters/symbols stimuli, we expected this manipulation to also affect perceptual/encoding time differences. That is, we expected the set size manipulation to also manifest in the non-decision time parameters. Given the motor speed manipulation should only serve to slow motor speed execution and not differentially enlist cognitive resources, we expected only a change in the non-decision time parameter as a function of the Spring Pressure manipulation. While boundary separation is not the primary focus of the present investigation, the intensity of the motor response may lead to slower responses in the stiff condition. This may manifest as an effect of motor response on boundary separation. Moreover, inasmuch as different set sizes reflect a task difficulty manipulation and task difficulty does not change boundaries (Mulder et al., 2013; Voss et al., 2004), as boundaries may be set by the participant a priori, we deemed it unlikely to see an effect of the set size manipulation on boundary separation. Although we note there seems to be limited research into this area so we leave this prediction unconstrained.

## Method

We present two experiments investigating the effect of motor slowing on DDM parameter estimates. All designs and procedures are identical across experiments except for the stimulus materials. Data for both experiments are available here: https://osf.io/w9s4q. This study received IRB approval. None of the experiments were preregistered.

### Participants

A prior investigation of the DDM that included a motor manipulation reported a sample size of *N* = 36 participants (Voss et al., 2004). Our sampling strategy in Experiment 1 was to post study timeslots for a few weeks in advance with the goal of collecting at least 36 participants, then ceasing recruitment of new participants. Our sample size ended up larger than 36 because we completed data collection of participants that were already signed up for the study at the time we closed new recruitment. Given evidence for motor speed effects in Experiment 1, we planned to recruit about the same number of participants in Experiment 2. Students participated for partial course credit or entry into a $100 gift card lottery (x5 gift card drawings) with *N* = 53 in Experiment 1 and *N* = 51 in Experiment 2. The experimental task malfunctioned for one participant in Experiment 1 and two participants in Experiment 2 and their data was not included as part of the final samples.

### Assessments

Commonly used clinical assessments of information processing speed^2^ and upper extremity functioning were administered to all participants in the same order prior to the experimental procedure. Inclusion of these measures afford the opportunity to assess convergent validity between DDM parameter estimates and more traditional measures of cognitive and motor speed, respectively.

#### Oral and written symbol digit modalities test

On the symbol digit modalities test (Smith, 2002), participants are presented with a grid of symbols on a single sheet of paper along with an answer key of symbol-digit pairings positioned at the top. Participants use the key to indicate the number from the key that matches the symbol in the grid in 90 s and their score is total number correct. Participants completed an oral (responses are stated orally) and written (responses are written below each symbol) version.

#### Nine-hole peg test

On the nine-hole peg test (Mathiowetz et al., 1985), participants repeatedly place and remove nine small pegs into holes on a board, as quickly as possible. Participants completed 5 trials for each hand and their score was the average time across all trials.

### Design

2 Spring Pressure (Stiff vs. Soft) X 2 set size (3 vs. 5) within participant design.

### Materials and apparatus

**Fig. 1:**
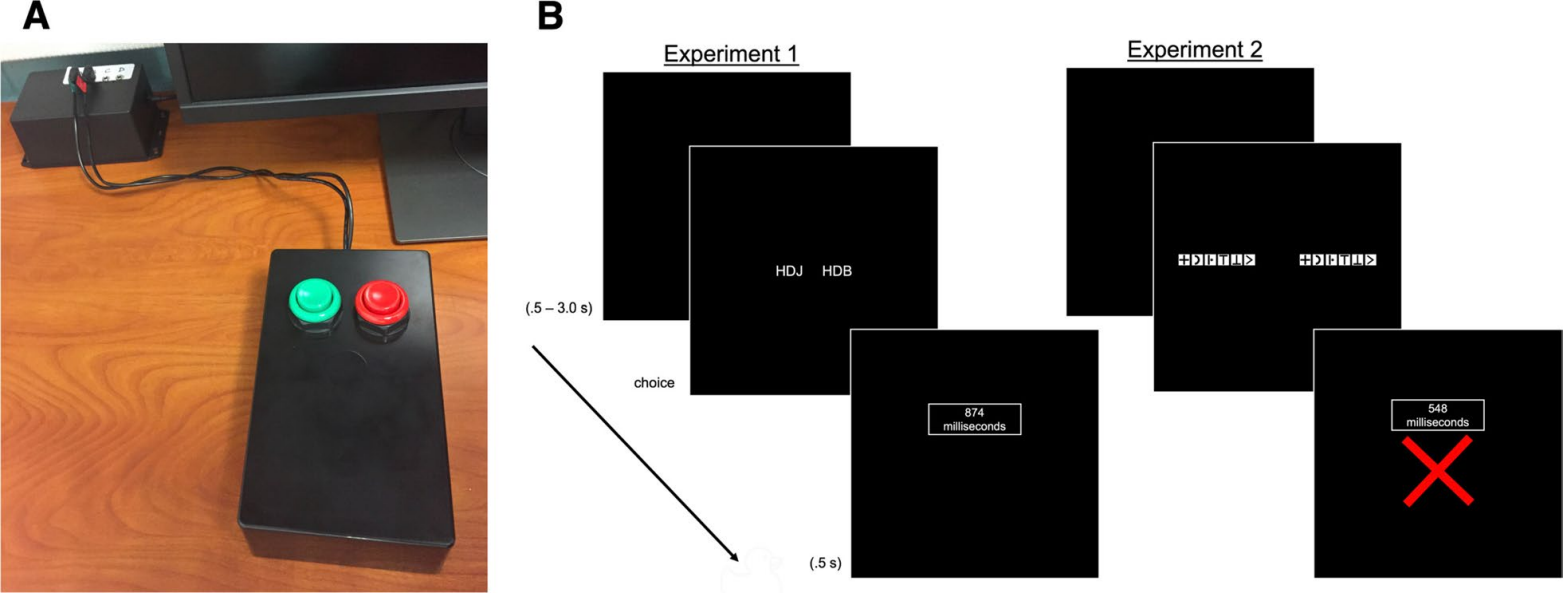
**A** Response button box and **B** figure timeline; variable inter-trial interval (0.5 to 3.0 s) was followed by a two-alternative forced choice letter (experiment 1) or pattern comparison (experiment 2) decision (3 vs. 6 set size) followed by feedback (0.5). Small set size and different (no match) trial with feedback for a correct response depicted for Experiment 1 and large set size same (match) trial with feedback for an incorrect response depicted for Experiment 2

Motor speed was manipulated by adjusting button spring pressure in custom-fabricated response button boxes with the following specifications (Fig. 1A). A custom-designed Adafruit ItsyBitsy 32u4–5 V 16 MHz printed circuit board main microcontroller and Texas Instruments logic gate connector (SN74AHC132D) were encapsulated inside of a rectangular box enclosure (4.8 in L × 3.2 in W × 4.8 in H), with two concave arcade-style button/microswitches. Response pressure was manipulated by using two different types of compression springs. Two different response boxes were used and interchanged over the course of the study. The response boxes were identical aside from the spring. One response box housed the stiff springs and the other response box housed the soft springs. The Soft spring pressure response box used standard springs (spring rate 0.37 lb/inch [0.04 N]). The Stiff spring pressure response box used replacement stiff compression springs (spring rate 11.4 lb/inch [1.3 N]) to simulate motor slowing. There was a total of 4 response boxes (2 stiff and 2 soft) which allowed us to test up to two participants at a time, in separate testing rooms.

### Procedures

Experiment 1 was a letter comparison task using English letters but excluding the 6 vowels AEIOUY to avoid word or non-word configurations across letter combinations or comparison by covert or overt verbalization. The letter comparison task is a closely matched computerized version of the paper and pencil letter comparison task (Salthouse, 1993; Salthouse & Babcock, 1991). Experiment 2 was a pattern comparison task that used 9 symbols designed to be similar to digit-symbol substitution tests used clinically. During the experimental task, a blank inter-trial-interval was randomly presented for between 0.5 and 3.0 s. This was followed by two letter/symbol strings either 3 or 5 characters long (set size manipulation) simultaneously presented on the left and right side of the screen (5.0 s). The strings matched on half of the trials and did not match on the other half of trials. Trials were presented in a randomized order and participants pressed the green button if the strings were the same (match) or red button if the strings were different (no-match). Feedback consisting of the participants’ response time and accuracy followed and remained on the screen for 0.5 s. The first 24 trials of each block were operationalized as practice leaving a total of 150 experimental trials per condition, for 600 total trials (Fig. 1B).

The order of the Spring Pressure manipulation was counterbalanced across 2 blocks. The research assistant set up the equipment prior to participant arrival. Participants were blind to condition, naïve to the purpose of the experiment and naïve to the Spring Pressure manipulation. After completing the first block of trials, participants were asked to take a brief break and wait outside of the testing room, at which point the research assistant swapped out the response box to be used in block 2, unbeknownst to participants. A small proportion of participants reported noticing the buttons “felt different” or were harder/easier to press (2/53 Experiment 1; 6/51 Experiment 2) and informally mentioned this to the research assistant at the end of the testing session—all participant-reported feedback was documented.

### Data preprocessing and exclusionary criteria

The first 24 practice trials from each block were discarded. Participants who performed at or below chance in a single condition (*N* = 0 Experiment 1; *N* = 1 Experiment 2) and participants whose attention and effort on the task could not be verified (operationalized as performances ≤ 2 standard deviations below group-level accuracy along with participants with abnormally short or long RTs, ≤≥ 2 standard deviations shorter/longer than the group-level mean; *N* = 5 Experiment 1; *N* = 3 Experiment 2) were excluded from all analyses. Response times shorter than 0.2 s or longer than 3 standard deviations above the participants mean were discarded. This resulted in removal of 0.69% of trials in Experiment 1 and 0.54% of trials in Experiment 2.

### Data analysis

#### Behavioral accuracy and response time

We use Bayes factors for ANOVAs and *t*-tests as our primary inferential approach to understanding changes in behavioral measures of accuracy and reaction times across conditions. Bayes factors are presented as the ratio between the probability of the data given an effect (alternative) to the probability of the data given no effect (null). In this context, a value of 7 in support of an effect should be interpreted as the alternative being 7 times more likely than the null. To calculate the Bayes factors for each main effect and interaction listed in the text we first found the best fitting model and then compared it to the equivalent model including or excluding each effect as appropriate. Bayes factors for each of the possible combinations of main effects and interaction are included in Table 1 relative to the null effects model. Bayes factors were calculated using the default Cauchy prior with a scale of *r* = 0.0707. All statistical analyses were computed using the BayesFactor package (Morey & Rouder, 2015) in R version 4.0.3.

**Table 1:** Bayes factor ANOVA statistics for behavioral accuracy and response time

	Accuracy	Response time
Experiment 1		
Spring pressure	4.19E+01	6.55E+02
Set size	5.72E+15	2.07E+33
Spring pressure + set size	1.62E+19	2.76E+45
Spring pressure + set size + spring pressure × set size	5.67E+18	5.63E+44
Experiment 2		
Spring pressure	2.74E+00	1.52E+00
Set size	1.27E+10	2.70E+38
Spring pressure + set size	1.32E+11	3.86E+41
Spring pressure + set size + spring pressure × set size	3.13E+10	8.46E+40

Listed values are relative to the null model (see, “Behavioral accuracy and response time” section in main text for additional clarification)

#### Hierarchical drift diffusion model parameter estimation

We implemented computational modelling with a Bayesian hierarchical drift diffusion model (HDDM) using the HDDM 0.6.0 Python toolbox (Wiecki et al., 2013) to evaluate differences across drift rate, boundary separation and non-decision time as a function of experimental manipulations of set size and spring pressure Condition. The hierarchical Bayesian implementation of the drift diffusion model allows for estimation and recovery of model parameters at the subject level and constrained by the group. We identified several theoretically meaningful combinations of drift rate, boundary separation, and non-decision time and fit each of these versions of the model with the corresponding parameters, *v*, *a*, and *Ter*, allowed to vary freely across conditions (see Table 2 for all models) using Markov Chain Monte Carlo simulation. Bias (*z*) and inter-trial variability parameters (*sv, st, sz*) were included in all models but constrained as group only node estimates given the challenges and uncertainty associated with estimating inter-trial variability parameters (Boehm et al., 2018; Wiecki et al., 2013). To ensure adequate posterior estimation we ran 5000 sampling iterations and discarded the first 1000 iterations of the chain as burn-in. We conservatively specified a 5% probability of outliers and assigned outliers to a uniform outlier distribution using the *p_outlier* command.

**Table 2:** Model fits for Experiments 1 and 2 and model selection using Bayesian Predictive Information Criterion (BPIC)

Model	Drift Rate (*v*)	Boundary separation (*a*)	Non-decision time (*Ter*)	Experiment 1		Experiment 2	
				BPIC	Difference from best model	BPIC	Difference from best model
Model 1 (full model)	SS, SPC	SS, SPC	SS, SPC	24,498	34	55,216	100
Model 2	SPC	SS, SPC	SS, SPC	25,111	647	55,510	394
Model 3	SS		SS, SPC	24,786	322	55,758	642
Model 4	SS	SS, SPC	SS, SPC	24,464	0	55,150	34
Model 5	SS	SS	SS, SPC	24,664	200	55,116	0
Model 6	SS	SPC	SS, SPC	24,592	128	55,765	649
Model 7	SS	SS, SPC	SS	24,697	233	55,474	358
Model 8	SS	SS, SPC	SPC	25,021	557	55,574	458
Model 9		SS, SPC		26,058	1594	56,492	1376

Table indicates parameter estimates that were allowed to vary for each model

*SS* set size, *SPC* spring pressure condition

#### Model selection

Trace and convergence chains were first visually inspected to verify model convergence. To verify that acceptable model fits were obtained we compared posterior predictive cumulative-distribution-function quantile plots to the empirical data for each participant fit (see https://osf.io/w9s4q). To determine the best model of how the manipulations affected cognitive processing, candidate models were compared using Bayesian Predictive Information Criterion (BPIC) (Ando, 2007) to evaluate goodness of model fit. BPIC combines the likelihood function with a penalty for model complexity to evaluate the fit of the model. Lower values indicate better fit. BPIC is advantageous in that it applies a more stringent correction than Deviance Information Criterion by using a larger penalty term for each additional model parameter.

#### Correlations

We computed the Pearson correlation between the HDDM drift rate and non-decision time parameter estimates (model 4, see below) to further evaluate the dependence/independence between these two estimates. Additionally, we correlated the HDDM parameter estimates with participants performances on both versions (oral and written) of the symbol digit modalities test and nine-hole peg test to evaluate convergent validity between the DDM parameter estimates and assessments commonly used in practice. Because the only difference between Experiments 1 and 2 was the type of stimulus materials used, we present the correlational analyses with data collapsed across Experiments 1 and 2 to increase power.

## Results and discussion

### Experiment 1: letter comparison

#### Accuracy

The evidence favored the model with 2 main effects, BF = 1.6X10^19^, with evidence against keeping the model with the interaction, BF = 0.35. Accuracy decreased with larger set sizes. Accuracy was higher in the Stiff in contrast to Soft Spring Pressure Condition (Table 1 and Fig. 1a).

#### Response time

**Fig. 2:**
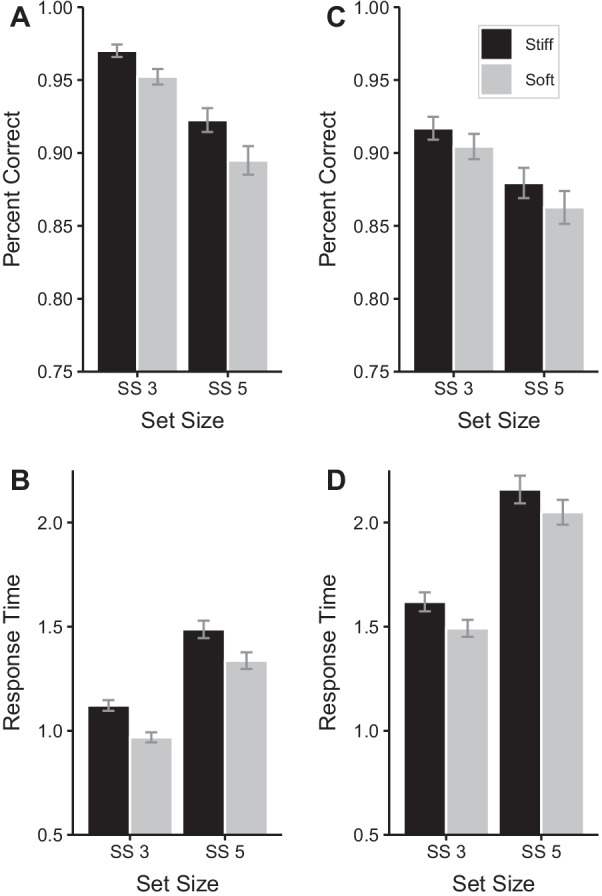
Mean accuracy and response time for Experiments 1 (**A**, **B**) and Experiment 2 (**C**, **D**). *SS* set size. Error bars are standard error of the mean

The evidence favored the model with 2 main effects, BF = 2.8X10^45^, with evidence against keeping the model with the interaction, BF = 0.20. Response times were longer in the larger set size condition. Response times were longer in the Stiff in contrast to the Soft Spring Pressure Condition (Table 1 and Fig. 2b).

#### HDDM model comparison

**Fig. 3:**
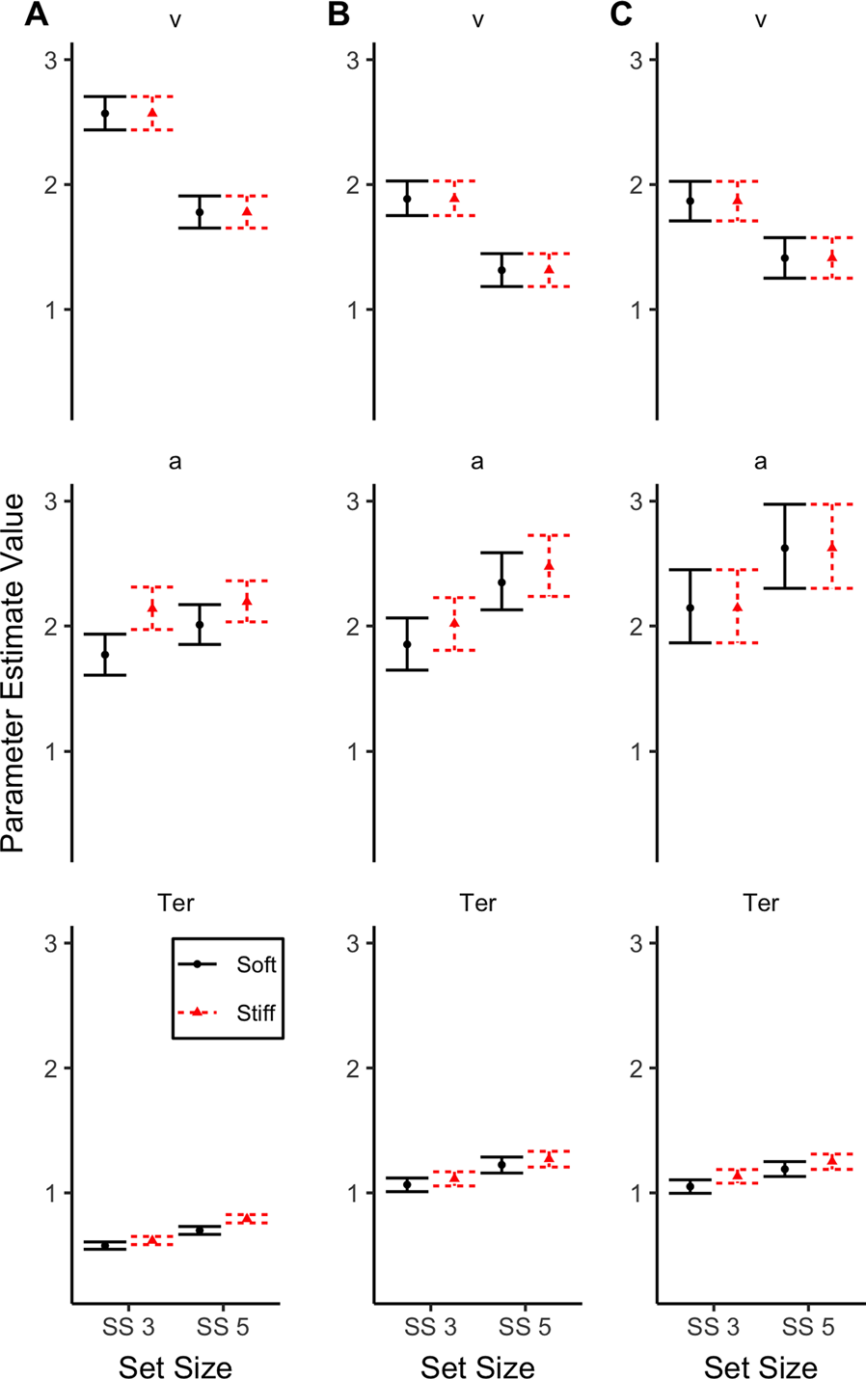
Mean parameter estimates (drift rate [*v*], boundary separation [*a*] and non-decision time [*Ter*]) for Experiment 1 model 4 (**A**) and Experiment 2 model 4 (**B**) and model 5 (**C**). *SS* set size. Error bars are 95% credible intervals

Table 2 lists the model fit indices in BPIC for all estimated models. Experiment 1 indicates two viable models with similar fits. The remaining models exhibit large increases in BPIC relative to the two best-fitting models. The two viable models are the full model (model 1) and the full model on boundary separation and non-decision time, but only an effect of set size on the drift rate (model 4). Model 4 had the better overall fit of the two models. Table 3 provides parameter estimates for Models 1 and 4 (see also, Fig. 3).

**Table 3:** Experiment 1 and 2 mean parameter estimates for Model 1 (full model on drift rate, boundary separation and non-decision time), Model 4 (full model on boundary separation and non-decision time, but only an effect of set size on the drift rate) and Model 5 (full model on non-decision time, but only an effect of set size on drift rate and boundary separation) and [2.5 to 97.5] quartiles for the posterior distributions

	Drift rate (*v*)		Boundary Separation (*a*)		Non-decision time (*Ter*)	
	SS 3	SS 5	SS 3	SS 5	SS 3	SS 5
Exp 1						
Model 1						
Soft	2.60 [2.45 to 2.75]	1.73 [1.60 to 1.87]	1.81 [1.65 to 1.97]	2.02 [1.85 to 2.19]	0.58 [0.55 to 0.61]	0.71 [0.67 to 0.74]
Stiff	2.57 [2.43 to 2.71]	1.86 [1.72 to 1.99]	2.16 [1.99 to 2.35]	2.26 [2.09 to 2.43]	0.62 [0.59 to 0.66]	0.80 [0.76 to 0.83]
Model 4						
Soft	2.57 [2.44 to 1.94]	1.78 [1.66 to 2.18]	1.78 [1.61 to 2.71]	2.01 [1.86 to 1.91]	0.58 [0.55 to 0.61]	0.70 [0.67 to 0.74]
Stiff	2.57 [2.44 to 2.32]	1.78 [1.66 to 2.37]	2.14 [1.98 to 2.71]	2.20 [2.04 to 1.91]	0.62 [0.59 to 0.66]	0.80 [0.76 to 0.83]
Exp 2						
Model 4						
Soft	1.89 [1.76 to 2.03]	1.32 [1.19 to 1.45]	1.86 [1.65 to 2.07]	2.35 [2.13 to 2.59]	1.07 [1.02 to 1.12]	1.23 [1.16 to 1.29]
Stiff	1.89 [1.76 to 2.03]	1.32 [1.19 to 1.45]	2.02 [1.81 to 2.23]	2.48 [2.24 to 2.73]	1.12 [1.06 to 1.17]	1.28 [1.21 to 1.34]
Model 5						
Soft	1.87 [1.71 to 2.03]	1.42 [1.26 to 1.58]	2.15 [1.87 to 2.46]	2.63 [2.31 to 2.98]	1.06 [1.00 to 1.11]	1.20 [1.14 to 1.26]
Stiff	1.87 [1.71 to 2.03]	1.42 [1.26 to 1.58]	2.15 [1.87 to 2.46]	2.63 [2.31 to 2.98]	1.14 [1.08 to 1.19]	1.26 [1.19 to 1.32]

*SS* set size

The difference between models 1 and 4 is that model 1 includes an effect of Spring Pressure on drift rate whereas model 4 does not. Given the similar BPIC values for both models 1 and 4, we verified that there was no effect of spring pressure on drift rate by inspecting the 95% credible intervals for drift rate parameters in model 1 (see Table 3). At each set size the 95% CIs for the stiff drift rate encompasses the mean soft drift rate. Similarly, the 95% CIs for the soft drift rate always encompasses the mean stiff drift rate. This provides converging evidence to the BPIC fits that drift rate is not affected by the Spring Pressure manipulation.

## Discussion

The primary findings from Experiment 1 demonstrate both the set size and spring pressure manipulation effectively change participants’ performances. Interestingly, we did find evidence that the Spring Pressure manipulation changed how accurate participants were, with more accurate responses in the Stiff in comparison with the Soft condition. The opposite pattern is evident in response time. The contrary effects across accuracy and response time in the Spring Pressure Condition implies a speed-accuracy trade-off induced by the response interface. One possibility is that the Stiff condition allowed participants to recover from incorrect responses. That is, if making a response and using the stiff button box, participants may have been able to rectify an incorrect button press and change to the correct response before fully depressing the response button. While the stiff spring pressure condition required more motor force to depress, it was more forgiving and allowed correction of errors. If this was the case, it elegantly explains higher accuracy in the stiff condition in comparison to the Soft condition.

HDDM estimation based upon the data from Experiment 1 indicate that drift rate is only affected by the cognitive set size manipulation while boundary separation and non-decision time parameters are affected by both cognitive and motor conditions. These data patterns support the *Independence Hypothesis* for drift rate and imply that the DDM and drift rate may be clinically useful for diseases with confounded motor and cognitive slowing. The effect of motor speed slowing on the cognitive boundary separation parameter is at first perplexing, but likely reflects the increased ability of participants to prevent accidental presses of the wrong button as reflected in the speed-accuracy trade-off interpretation above. This finding is in line with investigations reporting a motor contribution to the speed-accuracy trade-off with faster motor processes when under time pressure (Burle et al., 2014; Servant et al., 2015; Spieser et al., 2017; Weindel et al., 2021a). We discuss this in more detail in the “General discussion” section.

The letter stimuli in Experiment 1 were highly familiar letters. The nature of the letter stimuli deviated somewhat from more current clinical assessments that make use of less familiar stimuli (e.g., symbols). As a result, we changed the stimuli in Experiment 2 to symbols instead of letters, which we assume will reduce familiarity and increase the level of difficulty of the task. This stimulus change also brings the procedure more closely aligned to digit-symbol substitution tests used clinically. This change provides conceptual replication of Experiment 1 while simultaneously allowing us to evaluate convergent validity between the two modelling analyses across experiments. Because participants are highly familiar with letter-recognition (Krueger, 1975), the stimulus change from familiar letters to unfamiliar symbols should also change the amount of time needed to encode the information. This may manifest as larger non-decision times in Experiment 2. We reevaluate the findings of Experiment 1 but use these revised less familiar symbol stimuli in Experiment 2.

### Experiment 2: *pattern comparison*

#### Accuracy

The evidence favored the model with 2 main effects, BF = 1.3X10^11^, with evidence against keeping the model with the interaction, BF = 0.24. Accuracy decreased with larger set sizes. Accuracy was higher in the Stiff in contrast to Soft Spring Pressure Condition (Table 1 and Fig. 1c).

#### Response time

The evidence favored the model with 2 main effects, BF = 3.9X10^41^, with evidence against keeping the model with the interaction, BF = 0.22. Response times were longer in the larger set size condition. Response times were longer in the Stiff in contrast to Soft Spring Pressure Condition (Table 1 and Fig. 2d).

#### HDDM model comparison

Table 2 lists the model fit indices in BPIC for all estimated models. Experiment 2 indicates two viable models with similar fits, models 4 and 5. Model 5 had the better fit of the two. The remaining models exhibit large increases in BPIC relative to the two best-fitting models. The difference between models 4 and 5 is inclusion of the effect of Spring Pressure Condition on boundary separation for model 4 but not for model 5. In both models 4 and 5, there is no effect of Spring Pressure on drift rate (Table 2 and Fig. 3).

## Discussion

The critical pattern of findings in Experiment 1 were replicated in Experiment 2 with an alternative stimulus set, adding support for the *Independence Hypothesis* and use of the DDM and drift rate as a clinical estimate of “speed”. Although model fits differed slightly in Experiment 2, the difference was not relevant to the central theoretical focus of our analysis. Interestingly, the speed-accuracy trade-off under the Spring Pressure manipulation was also corroborated. This provides additional across experiment evidence for a speed-accuracy tradeoff when motor response is stiff. Because the same patterns emerged across experiments, we reserve detailed discussion of these replicated findings in the “General discussion” section.

### Across experiment correlational analyses

All correlations are presented in Table 4 and are computed using the parameter estimates from model 4, collapsed across experiments 1 and 2. Given our hypotheses were specific to (a) drift rate and non-decision time and (b) how these may relate to supplemental assessments (convergent validity) we focus primarily on interpreting these correlations.

**Table 4:** Correlations between symbol digit modalities test (SDMT), nine-hole peg test (NHPT) and HDDM parameter estimates for drift rate (*v*), boundary separation (*a*) and non-decision time (*Ter*)

	SDMT Oral	SDMT Written	NHPT	SS 3 *v*	SS 5 *v*	SS 3 Stiff *a*	SS 5 Stiff *a*	SS 3 Soft *a*	SS 5 Soft *a*	SS 3 Stiff *Ter*	SS 5 Stiff *Ter*	SS 3 Soft *Ter*	SS 5 Soft *Ter*
SDMT Oral													
SDMT Written	0.40**												
NHPT	− 0.13	− 0.17											
SS 3 *v*	0.35**	0.21*	− 0.11										
SS 5 *v*	0.32**	0.23*	− 0.05	0.84**									
SS 3 Stiff *a*	− 0.05	0.04	0.20	− 0.03	0.12								
SS 5 Stiff *a*	− 0.05	0.04	0.12	− 0.20	0.12	0.65**							
SS 3 Soft *a*	− 0.19	0.04	0.13	− 0.28**	0.00	0.71**	0.72**						
SS 5 Soft *a*	− 0.11	0.07	0.10	− 0.30**	0.06	0.55**	0.91**	0.74**					
SS 3 Stiff *Ter*	− 0.16	− 0.12	*0.06*	− 0.47**	− 0.38**	− 0.03	0.40**	0.14	0.37**				
SS 5 Stiff *Ter*	− 0.17	− 0.13	*0.06*	− 0.47**	− 0.42**	0.05	0.31**	0.12	0.29**	0.94**			
SS 3 Soft *Ter*	− 0.18	− 0.14	*− 0.03*	− 0.57**	− 0.45**	− 0.08	0.37**	0.12	0.40**	0.93**	0.87**		
SS 5 Soft *Ter*	− 0.21*	− 0.20*	*− 0.05*	− 0.57**	− 0.49**	− 0.06	0.30**	0.18	0.29**	0.89**	0.87**	0.93**	

Parameter estimates are derived from a hierarchical structure which may in some cases inaccurately estimate correlation strength. These values should be interpreted with a degree of caution

Italics values emphasize primary correlations of interest described in main text. *N* = 1 participant missing SDMT Oral and *N* = 1 participant missing NHPT not included in correlational analysis. Given model 4 was the preferred model from experiment 1 and similar BPIC values for models 4 and 5 from experiment 2, correlations are computed using model 4 parameter estimates combined across experiments 1 and 2

*SS* set size

#### Intercorrelation across HDDM parameter estimates

The drift rate parameter estimates for both set sizes 3 and 5 were negatively correlated with all non-decision time parameter estimates. This pattern was evident across all set size and spring pressure conditions (Table 4).

It is important to remember that non-decision time captures multiple elements; time to encode the representation (*Te*) *and* motor response execution speed (*Tr*) (Weindel et al., 2021a). The correlations reported here may reflect individual differences in general cognitive information processing ability. That is, participants who are faster at encoding (*Te*) would likely also likely be faster at evidence accumulation. The present experimental manipulations and modelling approach provide support that drift rate is independent of motor execution time (*Tr*). The correlation between parameters suggest that non-decision time may not be completely independent from drift rates, e.g., encoding and motor planning in the brain.

#### HDDM X supplemental assessments

The drift rate parameter was moderately positively correlated with both the oral and written version of the symbol digit modalities test. Specifically, participants who performed better on the symbol digit modalities test also had larger drift rates. This provides evidence that the symbol digit modalities test only has a small cognitive “speed” component (Average *r*^2^ = 0.08). There were no correlations between the nine-hole peg test and non-decision time parameters. Lack of a relationship provides support that the nine-hole peg test may be an independent measure of motor speed/coordination that does not overlap non-decision time.

## General discussion

One of the primary assumptions of the DDM is that drift rate is uncontaminated and independent of motor processes. Interestingly, the validity of this assumption has undergone little direct empirical evaluation. The veracity of this assumption is critical for both basic research and clinical investigations that aim to model latent cognitive processes involved in decision making. This is especially true in clinical research where terms like “processing speed” are attributed to multifarious measures and disease processes may lead to both cognitive and motor slowing. Cognitive and motor disease-related changes introduce a unique confound into psychometric measurement. Therefore, a measurement technique that dissociates motor and cognitive speed has strong potential as a clinical trial outcome measure. Across two experiments we manipulated motor speed by changing the amount of spring pressure required to depress the response key. Our findings provide empirical support for the *Independence Hypothesis*. That is, changes in motor speed cause changes in non-decision time, however, changes in motor speed do not cause changes to the drift rate. These data support the assumption that the drift rate is uncontaminated by motor speed slowing.

### Set size effects on DDM parameter estimates

While the primary purpose of the present investigation was to evaluate the influence of motor speed on the drift rate parameter, there are additional findings that are of theoretical interest. We first discuss these findings and then return to interpret the findings of the main aim in the next section. The outcome of the HDDM computational modelling demonstrates that drift rate is affected by the cognitive set size manipulation while boundary separation and non-decision time parameters are affected by both the cognitive and motor manipulations. We discuss each of the effects of set size on the DDM parameters in turn.

The effect of set size on drift rate suggests that drift rate is a sensitive measure of the rate of information accumulation that is impacted by the challenging nature of the task. Larger set sizes should require more cognitive resources to make comparative judgments, and this is evident in that larger set sizes had smaller drift rates while the small set size had larger drift rates. The effect of the set size manipulation was also evident for the boundary separation and non-decision time parameters. The nature of the set size effect on boundaries indicated higher boundaries for larger set sizes and this replicated across Experiments 1 and 2. This may be interpreted as larger set sizes requiring more information be accumulated before a response is initiated. The effect of set size on non-decision time likely reflects more time devoted to encoding and comparison processes with larger set sizes. This interpretation is further supported by a between experiment difference in non-decision times. With spring pressure held constant across experiments, the only difference came down to the nature of the stimuli used. Non-decision times were larger in Experiment 2 with less familiar symbol stimuli in contrast to the familiar letter stimuli used in Experiment 1. In sum, the set size manipulation manifests as an effect across all three HDDM parameters due to the multiple cognitive processing components that are required to make the cognitive decision. Crucially, the experimental manipulations and modelling suggest drift rate is independent from the influence of motor speed effects. Interestingly, the correlational analyses show evidence for moderate negative correlations between the drift rate and non-decision time parameters. Specifically, larger drift rates were related to smaller non-decision times. This is likely a necessary consequence of the experimental design and how limited cognitive resources would need to be distributed more widely across items in the higher compared to lower cognitive load conditions, set size 5 versus set size 3, respectively. Specifically, the division of resources across items would be reflected in a lower drift rate *and* a higher encoding time. That is, more items/information in set size 5 would require additional processing to develop a stable representation, resulting in a correlation between drift rates and non-decision times.

### Motor speed effects on DDM parameter estimates

A prerequisite for the present research was to effectively manipulate motor speed, something that there is mixed support for in past research. For example, some investigations report effects of experimental motor speed manipulations that lead to changes in non-decision time (Lerche & Voss, 2019; Voss et al., 2004), whereas other investigations report no effect of motor speed manipulations on non-decision time (Gomez et al., 2015; Weindel et al., 2021a). We designed custom response boxes that accommodated stiff springs and used a within participant manipulation. The data support the expected effect of motor speed slowing on non-decision time. This effect provides confirmatory evidence that the motor speed manipulation was successful. This is in contrast to Weindel et al. (2021a) who used a between experiment spring pressure manipulation and reported no changes in non-decision time. In that investigation, the difference between the soft and stiff springs was only 3 times larger in magnitude, 2 N versus 6 N, respectively. The relative difference between the soft and stiff spring in the present investigation was about 31 times larger in magnitude, 0.04 N g versus 1.3 N, respectively. The larger difference in the present investigation required more force to depress the response key in the stiff relative to soft condition. This difference likely maximized motor slowing into an observable effect.^3^ However, the absolute difference in Weindel et al. was 4 N while the absolute difference in the present investigation was 0.9 N and differences in magnitude may not completely account for the discrepant findings across studies. The within participant manipulation used presently would reduce between participant/experiment variability that may have obfuscated an effect in Weindel et al. (2021a). Together, the presence of an effect of the Spring Pressure manipulation on non-decision time and absence of this effect on drift rate is critical for interpretation of independence of motor speed on drift rates.

One auxiliary finding in the present research was a trade-off in speed and accuracy as a function of the Spring Pressure manipulation. Specifically, behavioral accuracy was higher in the stiff motor speed condition and behavioral response times were also longer. This performance trade-off replicated across Experiments 1 and 2. The outcome of the HDDM computational modelling also supported the finding with a clear effect in the boundary separation parameter for the Spring Pressure Condition in experiment 1 and some evidence for this same pattern in experiment 2 for model 4 but not model 5. The nature of the difference was wider boundaries for the Stiff condition that can be interpreted as more cautious responding in this condition. Initially, understanding why a motor speed manipulation would cause a change in response caution seems perplexing. However, this effect can be elegantly explained when considering the nature of responding. Specifically, errors may be more forgiving when the spring pressure was more difficult to depress. For example, consider a situation where the participant is presented with a no-match trial and they begin to initiate a “same” response out of habit or without properly considering the response. In this example, we can assume that the neural motor response signal would begin to initialize, and their finger may even begin to depress the response key. During this same time, the participant may recognize their “same” response is actually incorrect. Under the Stiff condition the button is more challenging to depress, allowing additional time to rebound and change the response to “different”. In the Soft condition, the button is far less challenging to depress and therefore, much less forgiving. This interpretation is congruent with earlier studies that demonstrated participants are able to detect and correct erroneous responses as they occur (Rabbitt & Vyas, 1981). Future research should use optimized experimental design parameters to directly evaluate this claim and estimate the individual level bias (*z*) DDM parameter. Another account whereby motor initiation occurs prior to reaching the response threshold may also help explain this pattern of findings.

One assumption of the DDM is that the motor response is initiated *only* after reaching the decision boundary (Ratcliff & McKoon, 2008; Ratcliff & Tuerlinckx, 2002; White et al., 2010). Standing in contrast to this assumption are studies decomposing “partial error” trials using EMG. The partial error finding is that incorrect response EMG activation (motor initiation) is followed by the correct response for a portion of trials (Burle et al., 2002, 2014; Coles et al., 1985). That is, the motor response may be initiated while the decision process is still ongoing (Weindel et al., 2021b). The pattern of EMG partial errors provides evidence against the motor response always commencing after the response threshold is reached with subthreshold motor events (EMG activity) evident during the decision process. In the context of the current research, it is possible that the speed accuracy tradeoff for the Spring Pressure Condition was related to a partial error. The more challenging button press in the Stiff condition (wider boundaries) may have provided an advantage whereby adjustment of an incorrect motor initiation was easier to override and correct in contrast to the lower spring pressure in the Soft condition. In-line with the present speed accuracy trade-off under the motor speed manipulation, other corroboratory evidence demonstrates a parallel finding to what we report here. That is, manipulating time pressure (i.e., manipulating the speed-accuracy trade-off) causes changes in non-decision times and specifically, motor speed (Tr) (Spieser et al., 2017; Steinemann et al., 2018; Weindel et al., 2021a). A future investigation incorporating EMG with a Spring Pressure manipulation similar to the current investigation will be informative for fully understand the change in boundaries observed presently. Moreover, changes in boundary separation related to motor slowing and partial errors may have applied or clinical implications.

### Applied and clinical implications

Interindividual heterogeneity within and across neurological diseases often results in differential rates of cognitive and motor slowing. Two patients may score the same under traditional behavioral assessment approaches that use either response time or accuracy. Decomposing behavioral responses using the DDM may reveal that the processes that underlie the low scores differ for the two patients. One patient may have a lower drift rate which would corroborate a general pattern of cognitive slowing while the other patient may have a change in boundary separation. The second patients’ performance may be driven by a speed accuracy trade-off and difficulty overriding partial errors as a result of motor slowing. Other combinations, for example, larger non-decision time or an interaction of changes across multiple DDM parameters are also possible. When evaluated traditionally, the treatment for patient one may be inappropriate to apply to patient two, given the underlying causes (e.g., lower drift rates vs. wider boundaries) are very different. Thus, there is strong prognostic potential for using the DDM clinically to decompose process-level cognitive differences. In support of this, older adults are typically slower than young adults and this was interpreted early on as generalized slowing of information processing speed (Myerson et al., 1992). In contrast to this explanation, contextualizing aging differences under a DDM framework showed that older adults are slower because of wider boundary separation and larger non-decision time. Interestingly, older adults’ drift rates do not differ from younger adults, providing some evidence against the slowing of information processing speed explanation (Ratcliff et al., 2006, 2007, 2010). Understanding the root cause of person-specific and disease-specific cognitive changes becomes increasingly complex when comorbid conditions (e.g., depression, anxiety), disease modifying therapies, and/or medications that impact cognition are also factored in. Clinical application of the DDM may unveil novel precision treatment approaches for what are likely different clusters of disease-related cognitive disability profiles. We may find that those profiles are easily characterized under a DDM computational model framework.

The correlational analyses support the imprecise nature of traditional digit-symbol processing speed assessments. Our findings show only small positive correlations between the widely used symbol digit modalities test and the drift rate parameter. Assuming that the latent drift rate parameter is a relatively process pure measure of the rate of information processing, this finding suggests that only a small proportion of the variance (average *r*^2^ = 0.08) measured by the symbol digit modalities test reflects a speeded component. Inasmuch as the drift rate parameter is a measure of processing speed, this leaves a considerable amount of variance unaccounted for. The weak to moderate relationship corroborates other research demonstrating processing speed inferences based exclusively on digit-symbol tests are largely invalid (Jaeger, 2018; Joy et al., 2003; Mui et al., 2022; Sandry et al., 2021; Treviño et al., 2021) and may more likely reflect multimodal integration (Sandry & Dobryakova, 2021). Low specificity does not undermine the value associated with a highly sensitive and quick screener afforded by digit-symbol assessments. This does underscore the importance of follow-up assessment with precision instruments (Sandry et al., 2021), of which, response time models may be one viable approach (Mui et al., 2022). What will be even more informative is widespread application of computational modelling approaches, including the DDM, to quantify disease-related cognitive changes (Evans & Wagenmakers, 2019; Forstmann et al., 2016; Ratcliff et al., 2016; White et al., 2010; Wiecki et al., 2015).

Unlike the moderate relationship between the symbol digit modalities test and the drift rate parameter, and in contrast to our expectations, we did not observe a reliable relationship between the nine-hole peg test and the non-decision time parameter. This may be because the nine-hole peg test is a wholistic measure of upper extremity function, finger dexterity and/or eye-hand coordination more so than it measures finger motor speed. Alternative measures of motor speed, for example, finger tapping, or EMG may reveal reliable correlations between measures of motor speed and the non-decision time parameter. In fact, despite no difference as a function of the between experiment response force manipulation, motor time measured with EMG is weakly correlated with non-decision time (Weindel et al., 2021a). Importantly, the participants in the present sample were healthy controls. It is reasonable that correlations in clinical samples, with less restricted ranges on the nine-hole peg test, may reveal more robust relationships.

One limitation of computing correlations between hierarchical Bayesian parameter estimates and behavioral responses (e.g., symbol digit modalities test and nine-hole peg test) is that including hierarchical derived estimates may lead to systematic underestimation of the population correlation (Katahira, 2016; Ly et al., 2017). As a result, the present correlational inferences should be considered with this in mind and treated with a degree of caution. While an important caveat to the correlational analysis, findings from one simulation study suggest that biased estimation may be less concerning with larger sample sizes (Katahira, 2016). In the present investigation our correlational inferences are based on sample data combined across both experiments. This large sample may offset biased estimation to some degree and this can be verified in follow-up research.

## Conclusion

Across two experiments, we evaluated a main assumption of the DDM. Specifically, we tested and found support for the hypothesis that differences in motor speed are not captured by the drift rate. That is, drift rate is experimentally independent from non-decision time, but non-decision time is not necessarily independent from manipulations that impact drift rate. The present findings provide critical empirical support that a computational modelling approach, the HDDM, has strong potential to serve as a precision measure of information processing speed in clinical populations who experience both cognitive and motor slowing.

## Data Availability

Data for both experiments are available here: https://osf.io/w9s4q.

## Footnotes

1 The methodology of Voss et al. (2004) is ambiguous with respect to the participants’ specific requirement in the non-handicap condition. We assume that participants positioned their left and right hands on the C and M keys. It is also possible that participants used two fingers from the same hand. In either case, the handicap condition required movement between keys, which we assume was not a requirement in the comparison non-handicap condition.

2 We use the label “information processing speed” here, in the general sense to remain consistent with the clinical literature. See the Introduction for details about the amorphous nature of this assessment and similar digit-symbol tests.

3 In a preliminary pilot experiment using a design similar to the present experiments, we changed the spring pressure in mechanical keyboards (0.004 N vs. 0.018 N). We found no effect of the Spring Pressure manipulation given the small difference in spring pressure, only 4.11 times difference in magnitude. This is in-line with no observable effect of reported in Weindel et al. (2021a, 2021b) and guided our decision to build custom response boxes that would accommodate stiffer springs, thereby maximizing the size of the effect.
